# Predicting New Daily COVID-19 Cases and Deaths Using Search Engine Query Data in South Korea From 2020 to 2021: Infodemiology Study

**DOI:** 10.2196/34178

**Published:** 2021-12-22

**Authors:** Atina Husnayain, Eunha Shim, Anis Fuad, Emily Chia-Yu Su

**Affiliations:** 1 Graduate Institute of Biomedical Informatics College of Medical Science and Technology Taipei Medical University Taipei Taiwan; 2 Department of Mathematics Soongsil University Seoul Republic of Korea; 3 Department of Biostatistics, Epidemiology, and Population Health Faculty of Medicine, Public Health, and Nursing Universitas Gadjah Mada Yogyakarta Indonesia; 4 Clinical Big Data Research Center Taipei Medical University Hospital Taipei Taiwan

**Keywords:** prediction, internet search, COVID-19, South Korea, infodemiology

## Abstract

**Background:**

Given the ongoing COVID-19 pandemic situation, accurate predictions could greatly help in the health resource management for future waves. However, as a new entity, COVID-19’s disease dynamics seemed difficult to predict. External factors, such as internet search data, need to be included in the models to increase their accuracy. However, it remains unclear whether incorporating online search volumes into models leads to better predictive performances for long-term prediction.

**Objective:**

The aim of this study was to analyze whether search engine query data are important variables that should be included in the models predicting new daily COVID-19 cases and deaths in short- and long-term periods.

**Methods:**

We used country-level case-related data, NAVER search volumes, and mobility data obtained from Google and Apple for the period of January 20, 2020, to July 31, 2021, in South Korea. Data were aggregated into four subsets: 3, 6, 12, and 18 months after the first case was reported. The first 80% of the data in all subsets were used as the training set, and the remaining data served as the test set. Generalized linear models (GLMs) with normal, Poisson, and negative binomial distribution were developed, along with linear regression (LR) models with lasso, adaptive lasso, and elastic net regularization. Root mean square error values were defined as a loss function and were used to assess the performance of the models. All analyses and visualizations were conducted in SAS Studio, which is part of the SAS OnDemand for Academics.

**Results:**

GLMs with different types of distribution functions may have been beneficial in predicting new daily COVID-19 cases and deaths in the early stages of the outbreak. Over longer periods, as the distribution of cases and deaths became more normally distributed, LR models with regularization may have outperformed the GLMs. This study also found that models performed better when predicting new daily deaths compared to new daily cases. In addition, an evaluation of feature effects in the models showed that NAVER search volumes were useful variables in predicting new daily COVID-19 cases, particularly in the first 6 months of the outbreak. Searches related to logistical needs, particularly for “thermometer” and “mask strap,” showed higher feature effects in that period. For longer prediction periods, NAVER search volumes were still found to constitute an important variable, although with a lower feature effect. This finding suggests that search term use should be considered to maintain the predictive performance of models.

**Conclusions:**

NAVER search volumes were important variables in short- and long-term prediction, with higher feature effects for predicting new daily COVID-19 cases in the first 6 months of the outbreak. Similar results were also found for death predictions.

## Introduction

COVID-19 is a new disease entity that has caused a global pandemic, with more than 200 million cases and 4.5 million deaths since it was first reported at the end of December 2020 [1]. In contrast to the previous outbreaks of SARS and Middle East respiratory syndrome (MERS) that spread in clustered countries, COVID-19 exhibited massive disease transmission and longer periods of spread, even with implementation of multiple public health measures. Given this situation, predictions could greatly help in health resource management [2], particularly in terms of human resources and medical equipment deployment [3], as well as in preparing for upcoming future waves [4]. This approach will be beneficial for policy makers and health care managers [2], both in national government and at the level of local authorities [3].

However, as a new entity, COVID-19’s disease dynamics seemed difficult to predict [5]. Most existing COVID-19 prediction models are highly dependent on confirmed cases, which may lag behind underlying infections [6]. The number of confirmed cases may only represent the number of people who have sought medical attention due to the occurrence of moderate to severe symptoms [5]. Therefore, external factors need to be included in models to increase the accuracy of these models.

One of the most common emerging external variables included in COVID-19 prediction models is comprised of internet search data. These data are collected during information-seeking activities on Google, NAVER, Daum, Baidu, and other search engines. Studies used information-seeking activities are part of infodemiology studies. The term “infodemiology” was first introduced by Eysenbach [7] in 2002 as an acronym of information epidemiology. This field aimed to analyze online information in terms of its distribution and determinants for public health–related purposes [8]. In addition, infodemiology is a fast-growing field of research that can be assessed both from demand- and supply-side studies [9]. Search engine query data in infodemiology studies are used in demand-based studies, which may have several advantages in the case of the COVID-19 pandemic. An increase in search data has commonly preceded traditional COVID-19 metrics [5,10,11]; as such, these data may provide a real-time indication of symptoms in a population [6]. Therefore, constructed models could possibly detect new waves or peaks at an earlier stage of the outbreak [5].

A study by Rabiolo et al [12] found that models that included search data performed better than those that did not include the search volumes in the first month of outbreak prediction. Similar findings were also shown in two previous analyses—one studying data from Iran [13] and another studying data from India, the United States, and the United Kingdom [14]—for periods of 1 month and 3 months, respectively, after the first case was detected. However, other studies conducted in the United States demonstrated low accuracy in model prediction [15] and variability in model performances among states and time periods [16]. Both of those studies were constructed using time series data of less than 2 months. Hence, it remains unclear whether models incorporating online search volumes will lead to better predictive performances for longer periods of prediction of new daily COVID-19 cases and deaths. In this study, we assessed the predictive performance of NAVER search volumes at different pandemic stages in South Korea. Data were aggregated into four subsets: 3, 6, 12, and 18 months after the first case was reported. In brief, this study aimed to analyze whether search engine query data constitute an important variable for inclusion in models for short- and long-term prediction of new daily COVID-19 cases and deaths.

## Methods

### Data Sets

**Figure 1:**
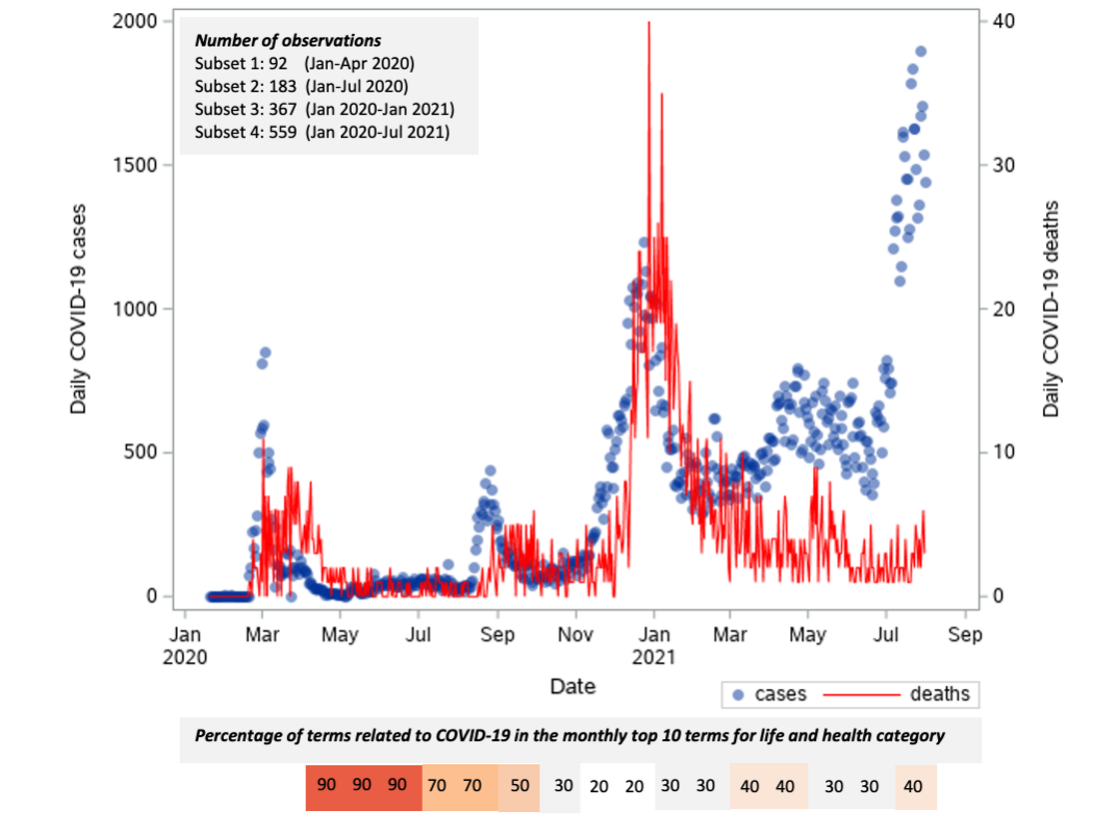
Time series of new daily COVID-19 cases and deaths in South Korea from January 20, 2020, to July 31, 2021. The information at the bottom of the figure describes the percentage of terms related to COVID-19 per month, from April 2020 to July 2021, out of the monthly top 10 terms for the life and health category (N=10). The list of terms is provided in Multimedia Appendix 1.

In this study, we used country-level case-related data, NAVER search volumes, and mobility data from Google and Apple. NAVER search volumes were retrieved from NAVER’s website [17] using terms related to COVID-19 and popular terms as of July 31, 2021. Terms in Korean, followed by their English translation, included the following: 코로 나 바이러스 (coronavirus), 코로나 바이러스 테스트 (coronavirus test), 메르 스 (MERS), 마스크 (face mask), 사회적 거리두기 (social distancing), 신천지 (Shincheonji), kf94 마스크 (kf94 mask), 일회용 마스크 (disposable mask), 온도계 (thermometer), 손 소독제 (hand sanitizer), 마스크스트랩 (mask strap), and Kf80 마스크 (kf80 mask). NAVER search volumes were queried in the Korean language, with quotation marks used for terms with more than two words, for all types of searches, genders, and age groups. Mobility data were collected from Google’s Community Mobility Reports [18] and Apple’s Mobility Trends Reports [19]. In addition to case-based data, daily cumulative COVID-19 cases and deaths were downloaded from the country-level time series data repository from the Center for Systems Science and Engineering at Johns Hopkins University [20]. A detailed description of all data used in this study is given in Table 1. Case-related data were retrieved from January 20, 2020—when the first COVID-19 case was reported in South Korea—to July 31, 2021. NAVER search volumes and mobility data were queried with a lag of 3 days to include more-recent observations in the analysis. Data were then aggregated into four subsets: 3, 6, 12, and 18 months after the first case was reported (Figure 1). Moreover, we also retrieved the monthly top 10 terms in the life and health category from NAVER beginning in April 2020 (Multimedia Appendix 1).

### Statistical Analysis

Explanatory variables (Table 1 [21]) were used to develop models for predicting new daily COVID-19 cases and deaths. The first 80% of the data in all subsets were used as the training set, and the remaining data served as the testing set. In order to determine the best-fitting model in each subset, generalized linear models (GLMs) with three different distributions (ie, normal, Poisson, and negative binomial) were developed, along with linear regressions (LRs) with lasso, adaptive lasso, and elastic net regularization.

**Table 1:** Data set description.

Data set^a^	Data description	Use
Case-based data	Daily cumulative cases and deaths; used to calculate new daily cases and deaths	Time series graph, correlation, and prediction analysis
Google Community Mobility data	Daily changes in time spent in six categorized places—retail and recreation, grocery and pharmacy, parks, transit stations, workplaces, and residential areas—compared to baseline days; median value from January 3 to February 6, 2020	Correlation and prediction analysis
Apple Mobility Trends data	Daily relative volume of direction requests, in driving and walking situations, in Apple Maps compared to a baseline volume on January 13, 2020	Correlation and prediction analysis
NAVER search volumes	Daily online searches made through NAVER search engines; data ranged from 0 to 100; queries were made based on 12 terms used in our previous study [21] and popular terms related to COVID-19 as of July 31, 2021, from the life and health category; data were retrieved using terms in the Korean language, with quotation marks used for terms of more than two words, for all types of searches, genders, and age groups	Correlation and prediction analysis

^a^All data sets include country-level data.

All analyses and visualizations were conducted using SAS Studio, which is part of SAS OnDemand for Academics (SAS Institute Inc). For the GLMs, proc hpgenselect in SAS was used to develop and test the model performance with stepwise selection and an α level of .05 in selecting variables for the model. Only statistically significant variables (*P*<.05) were included in the model. Furthermore, proc glmselect in SAS was used to construct LR models with steps of 25 and with the lowest Akaike information criterion (AIC) values in defining model selection. The 25 model construction steps were chosen in order to provide sufficient steps to define the best model with the lowest AIC value. Root mean square error (RMSE) values were defined as loss functions to assess the performance of models in the four subsets..

## Results

### Characteristics of New Daily COVID-19 Cases, Deaths, Mobility, and Search Data

The first case of COVID-19 in South Korea was reported on January 20, 2020, as shown in Figure 1. In the first 3 months of the outbreak, the mean number of new daily cases was 116.02. During this period, massive numbers of coronavirus tests were conducted along with the strict implementation of the social distancing policy. On February 7, 2020, the first coronavirus test kit was approved [22], and the first coronavirus drive-through test center was opened on February 23, 2020 [23]. The curve of cases was flattened, which led to an easing of social distancing rules at the national level beginning on May 6, 2020. A contact tracing system called KI-Pass was also introduced during this period [24]. Thus, with implementation of strict public health measures, the average new daily cases in the first 6 months of the outbreak dropped to 75.50, which was lower than that in the first 3 months.

However, a surge of cases occurred in mid-August, which led to a reinstating of level 2 restrictions beginning on August 28, 2020, in conjunction with mandatory mask-wearing. On October 12, 2020, restrictions were eased throughout most of the country, although a huge surge of cases developed as of mid-November. Level 2 restrictions were then tightened again [24]. This wave of cases remained high until the early months of 2021.

The first COVID-19 vaccine in South Korea was rolled out on February 28, 2021 [25]. Through the end of May, more than 700,000 people were newly vaccinated each day, but this number began to decrease by the end of June [26]. In early July, only around 1665 people were being vaccinated each day [27]. During this period, an immense wave of cases occurred that led to implementation of level 4 social distancing rules for the greater Seoul area beginning on July 26, 2021 [28]. The number of cases during this wave was larger than that of the other waves since the first reported COVID-19 case in South Korea. Time series analyses of new daily COVID-19 cases showed that implementation of public health measures heavily impacted the progression of cases. The number of new daily deaths seemed to follow the dynamics of COVID-19 cases, which were relatively higher in the third wave and lower in the fourth wave.

During the four waves of COVID-19 cases in South Korea, searches using various terms related to COVID-19 were captured in the NAVER database. In Figure 1, percentages of terms related to COVID-19 in the life and health category are presented. Due to the limitations of data querying in NAVER, retrospective top searches are only displayed starting from April 2020. A list of the top monthly terms is provided in Multimedia Appendix 1.

Figure 1 shows that a high percentage (9/10, 90%) of COVID-19–related terms were used in searches until June 2020. Afterward, the percentages decreased in the remaining months, with the lowest percentage (2/10, 20%) in November and December 2020. Relatively constant percentages of 30% (3/10) to 40% (4/10) of COVID-19–related terms used in internet searches were found in 2021. These findings reveal a massive use of COVID-19–related terms during online information-seeking activities in the early phase of the outbreak and a decreased pattern during the longer periods of the outbreak. In addition, top searches were mostly related to face masks, along with thermometers in April 2020 and hand sanitizers in August and September 2020.

**Figure 2:**
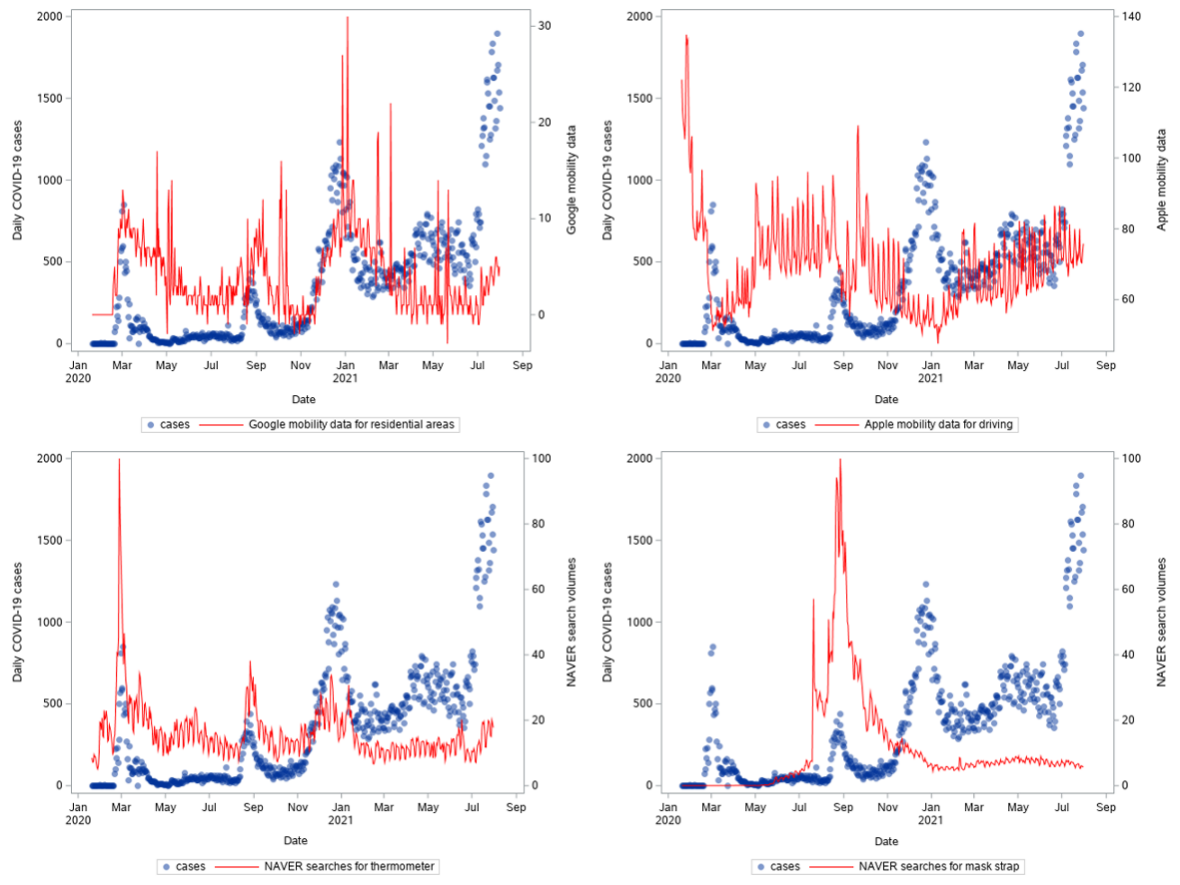
Time series of new daily COVID-19 cases, mobility data (top plots), and NAVER searches (bottom plots) in South Korea from January 20, 2020, to July 31, 2021.

**Figure 3:**
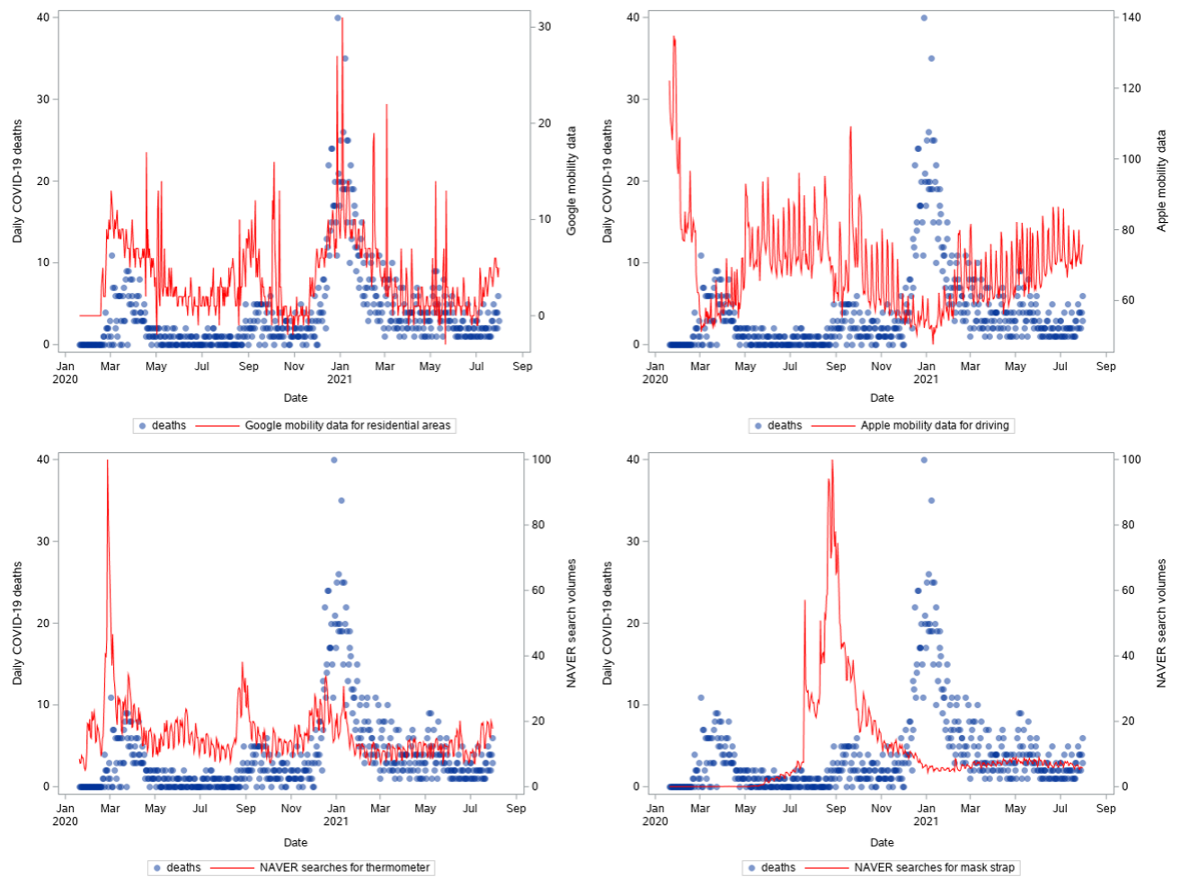
Time series of new daily COVID-19 deaths, mobility data (top plots), and NAVER searches (bottom plots) in South Korea from January 20, 2020, to July 31, 2021.

Furthermore, decreased trends of mobility captured by Google were found to resemble the dynamics of cases and deaths (Figures 2 and 3). This differed from Apple mobility data, which seemed to be higher in the first and second waves and increased as the fourth wave developed. Moreover, increased numbers of searches seemed to precede the surge in cases and deaths.

### Correlations of New Daily COVID-19 Cases and Deaths With Explanatory Variables in the Training Sets

In the early stages of the outbreak, regarding subsets 1 and 2, new daily cases in the last 3 days (*r*=0.75, *r*=0.83), Google mobility data (retail and recreation: *r*=–0.82, –0.72; transit stations: *r*=–0.79, *r*=–0.70; residential areas: *r*=0.80), Apple mobility data (driving: *r*=–0.73; walking: *r*=–0.72), and NAVER search volumes (face mask: *r*=0.75; Shincheonji: *r*=0.83; thermometer: *r*=0.83, *r*=0.70) were highly correlated with new daily COVID-19 cases (Multimedia Appendix 2). In the third and fourth subsets, high correlations were only found between new daily COVID-19 cases and new daily cases in the last 3 days (*r*= 0.85, *r*=0.93). Moreover, moderate correlations were found between new daily COVID-19 cases in the third subset and Google mobility data (retail and recreation: *r*=–0.53) and between new daily COVID-19 cases in the last subset and new daily deaths in the last 3 days (*r*=0.62), Apple mobility data (driving: *r*=–0.62), and NAVER search volumes (disposable mask: *r*=–0.55). Negative correlations were mostly found between new daily COVID-19 cases and mobility data, which showed a decrease in the public’s mobility during the pandemic period, particularly in the early stage of the outbreak. Negative correlations were also observed between new daily COVID-19 cases in the last subset of NAVER search volumes.

For new daily COVID-19 deaths, high correlations were only found for Apple mobility data (driving: *r*=–0.72; walking: *r*=–0.73) and NAVER search volumes (social distancing: *r*=0.72) in the first subset, and new daily cases in the last 3 days (*r*=0.71) and deaths (*r*=0.72) in the last subset. Similar to results in new daily COVID-19 cases, most of the negative correlations were found in mobility data in all subsets and NAVER search volumes in the last subset. Only Google mobility data for residential areas were positively correlated in all sets with both new daily COVID-19 cases and deaths. Results of the correlation analysis showed that higher correlations tended to be found in case-based data as the outbreak progressed, while reverse findings were found in mobility and internet search data.

### Model Performance

GLMs with a Poisson distribution performed better as compared to the other models in predicting new daily COVID-19 cases in the first subset (Table 2). This finding suggests that at the early stage of the COVID-19 outbreak in South Korea, new daily cases more closely resembled a Poisson distribution. Later, in the second subset, the distribution of cases tended to be normally distributed, leading to a GLM with a normal function becoming the best performing model. GLMs with Poisson and negative binomial distributions resulted in larger RMSE values, which suggest that the distribution of cases in this subset did not follow those distributions that tended to be skewed.

**Figure 4:**
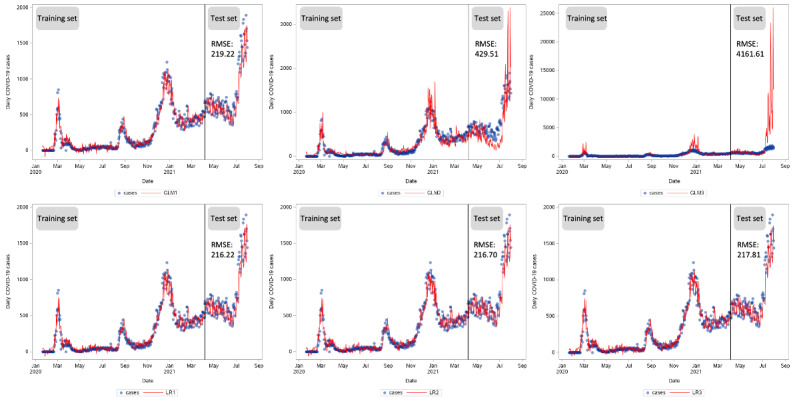
Time series of new daily COVID-19 cases in South Korea from January 20, 2020, to July 31, 2021, and predicted values in the generalized linear models (GLMs) and linear regression (LR) models. GLM1: GLM with a normal distribution; GLM2: GLM with a Poisson distribution; GLM3: GLM with a negative binomial distribution; LR1: LR model with lasso regularization; LR2: LR model with adaptive lasso regularization; LR3: LR model with elastic net regularization; RMSE: root mean square error.

**Figure 5:**
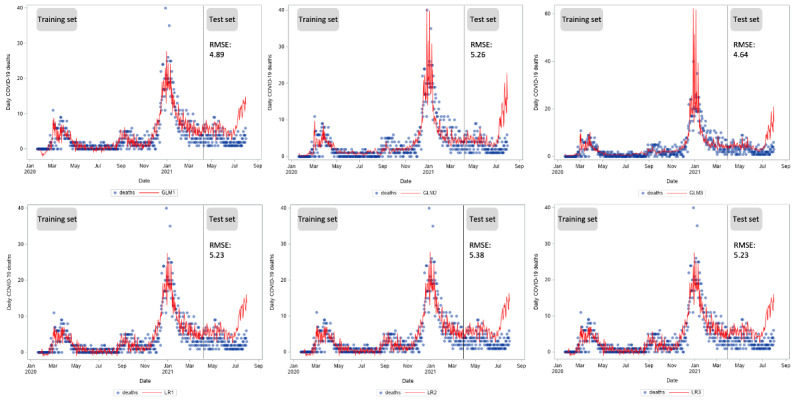
Time series of new daily COVID-19 deaths in South Korea from January 20, 2020, to July 31, 2021, and predicted values in the generalized linear models (GLMs) and linear regression (LR) models. GLM1: GLM with a normal distribution; GLM2: GLM with a Poisson distribution; GLM3: GLM with a negative binomial distribution; LR1: LR model with lasso regularization; LR2: LR model with adaptive lasso regularization; LR3: LR model with elastic net regularization; RMSE: root mean square error.

In the third and fourth subsets, the LR without regularization (GLM1) and the LR with regularization (LR1-3) performed very similarly (Figure 4). This finding shows that GLMs performed better in the first 6 months of the outbreak. Over a longer period, LR models with regularization outperformed the GLMs. In addition, better performance of the model was found in predicting new daily deaths compared to new daily cases (Figures 4 and 5). For death predictions, the best performing models were the GLM with a negative binomial function in the first, second, and fourth subsets, and the LR with adaptive lasso regularization in the third subset.

**Table 2:** Assessment of the performance of the models.

Model			Subset 1^a^, RMSE^b^					Subset 2^a^, RMSE					Subset 3^a^, RMSE					Subset 4^a^, RMSE		
			Training set		Test set		Training set			Test set		Training set			Test set		Training set			Test set
**Predictions of new daily COVID-19 cases**																				
	GLM1^c^	62.22		66.92		53.04			32.70^d^		48.01			378.94		85.75			219.22	
	GLM2^e^	43.71		29.29^d^		36.80			569,037.92		48.19			495.88		120.76			429.51	
	GLM3^f^	982.42		587.65		329.49			8,247,155.77		184.59			543.20		330.15			4161.61	
	LR1^g^	58.57		60.17		50.90			44.92		48.20			373.58		85.09			216.22^d^	
	LR2^h^	56.88		79.57		49.41			78.32		48.00			366.19^d^		84.52			216.70	
	LR3^i^	56.51		69.13		50.90			44.92		48.20			373.58		84.42			217.81	
**Predictions of new daily COVID-19 deaths**																				
	GLM1	3.10		4.89		2.52			1.04		2.08			6.79		2.80			4.89	
	GLM2	3.24		5.52		2.71			0.47		2.23			7.65		2.82			5.26	
	GLM3	3.25		3.79^d^		2.72			0.19^d^		2.24			17.02		3.81			4.64^d^	
	LR1	3.05		4.95		2.62			1.71		2.16			5.21		2.75			5.23	
	LR2	3.04		4.50		2.61			0.70		2.19			4.82^d^		2.75			5.38	
	LR3	3.05		4.95		2.62			1.71		2.16			5.23		2.75			5.23	

^a^Subsets 1 to 4: 3, 6, 12, and 18 months after the first case was reported in South Korea, respectively.

^b^RMSE: root mean square error.

^c^GLM1: generalized linear model with a normal distribution.

^d^The lowest RMSE value in the test subset.

^e^GLM2: generalized linear model with a Poisson distribution.

^f^GLM3: generalized linear model with a negative binomial distribution.

^g^LR1: linear regression model with lasso regularization.

^h^LR2: linear regression model with adaptive lasso regularization.

^i^LR3: linear regression model with elastic net regularization.

### Feature Effects

In terms of the importance of the variables, NAVER search volumes produced higher parameter estimates in the models compared to case-based variables, Google mobility variables, and Apple mobility variables in the first and second subsets (Multimedia Appendix 3) in predicting new daily COVID-19 cases. This finding infers that NAVER search volumes might have affected the model performances to a greater extent and illustrates the usefulness of those variables, particularly searches for “thermometer” and “mask strap.” However, parameter estimates of NAVER search volumes tended to have decreased in the third and fourth subsets. Higher parameter estimates were found in Google mobility data (ie, residential areas, transit stations, and workplaces) along with Apple mobility data (ie, driving) and case-based data (ie, new daily deaths in the last 3 days).

In this study, inclusion of NAVER searches for “thermometer” in models with longer periods seemed to be beneficial. In addition, negative values of parameter estimates were found for most of the mobility data, except for the residential type, in all subsets. Negative parameter estimate values were also found in NAVER searches for “coronavirus,” “coronavirus test,” “MERS,” “face mask,” “kf80 mask,” “disposable mask,” “Shincheonji,” and “hand sanitizer” in the third and fourth subsets.

In contrast to the abovementioned results, predictions of new daily deaths showed similar values of parameter estimates for case-based variables, mobility data, and NAVER search volumes (Multimedia Appendix 4). The numbers of variables included in the model were relatively fewer in the first and second sets compared with that in the third and fourth sets. However, most of the NAVER search volume variables were still included in the model, even in the last subset. Negative parameter estimate values were found for most of the mobility data, except workplace and residential types, in all subsets. Similarly, negative parameter estimate values for NAVER searches were also found in all sets. However, positive parameter estimate values were seen in all sets for case-based variables.

## Discussion

### Principal Findings

This study demonstrated an easy and explainable approach for determining the predictive performance of NAVER search volumes in four different subsets: 3, 6, 12, and 18 months after the first case was reported in South Korea. Subsets were used to create scenarios to analyze whether search engine query data are important variables for inclusion in models for short- and long-term prediction. In this study, we found that NAVER search volumes were useful variables in predicting new daily COVID-19 cases and deaths, particularly in the first 6 months of the outbreak. For longer prediction periods, NAVER search volumes were still found to constitute an important variable, although with a lower feature effect. In addition, this study discussed the role of search engine query data in infodemiology studies during the COVID-19 pandemic.

### Short- and Long-Term Predictive Performances

Findings exhibited massive use of COVID-19–related terms for information-seeking activities at the early stage of the outbreak, which decreased over the longer period of the outbreak. This indicated a huge surge in information searches in the early months of the outbreak, as only limited COVID-19–related information was circulating. However, in later periods, extensive amounts of information were available, such as online news and reports by health experts [29]. Thus, these induced decreases in online information-seeking practices, which were observed from search term use. Beginning in April 2020, top searches were mostly related to face masks (Multimedia Appendix 1). A previous study in South Korea [21] showed increased in searches for various keywords concerning national and international events in the first 2 months of the pandemic. Similar results were also found in a worldwide setting [10], Taiwan [30], the Philippines [31], and the United States [32,33]. In addition, changes in the use of terms might indicate public concerns throughout the pandemic stages. In the case of South Korea, searches tended to be more related to logistical needs, including face masks, thermometers, and hand sanitizers for certain months in the longer period of the pandemic.

In terms of the correlation analyses, negative correlations were observed in the last subset for NAVER search volumes, which demonstrated a decline of searches as the number of cases increased. This finding is in line with an earlier study [16]. Moreover, lower correlation coefficients were found in search data as the outbreak progressed. This indicated the public’s concern in terms of online information searches related to the ongoing outbreak, which tended to change over time. In addition to the prediction models, GLMs with different types of distribution functions may have been beneficial in predicting new daily COVID-19 cases and deaths in the early stages of the outbreak. Nonnormal distributions of cases and deaths could be better predicted using a Poisson or negative binomial function. Over a longer period, as the distribution of cases and deaths changed more into a normal distribution, LR models with regularization may have outperformed the GLMs. The use of regularization could also be important in preventing overfitting due to increased numbers of possible terms used in the longer period of prediction. This study also found that better performances of models were achieved in predicting new daily deaths compared to new daily cases, as found in a previous study [34]. This finding suggests higher variability of time series components (ie, trend, seasonality, and error) in new daily COVID-19 cases, which affected the prediction performances.

Furthermore, feature effects in the models showed that NAVER search volumes were useful variables in predicting new daily COVID-19 cases, particularly in the first 6 months of the outbreak. Searches related to logistical needs, particularly for “thermometer” and “mask strap,” showed higher feature effects in that period. Compared to previous studies [12-14], terms with higher feature effects in the models were varied, from COVID-19–related terms, symptoms, and preventive measures. For longer prediction periods, NAVER search volumes were still found to be important variables, although with lower feature effects demonstrated from values of the parameter estimates. This result suggests that term use should be considered to maintain the prediction performance. This task may be subject to several challenges, since terms selected from top searches might not always perform as important variables in the model. Therefore, extensive keyword queries are needed to ensure that all possible and related terms are included in the model development.

Lastly, NAVER search volumes were also found to be beneficial in predicting new daily COVID-19 deaths, even for longer periods. Negative parameter estimate values for NAVER searches in the models were in line with results of the correlation analyses. This possibly suggests a decline in searches as the number of cases increases, although NAVER search engine query data were still regarded as useful variables for inclusion in the models.

### The Role of Internet Search Data in Infodemiology Studies of COVID-19

As the COVID-19 pandemic has emerged, infodemiological studies related to COVID-19 grew exponentially. In general, such studies can be divided into three major subjects: studies to understand community online search behaviors, preliminary studies to assess possible use of search data for prediction purposes, and prediction analyses. Studies to understand community online search behaviors are mostly aimed at assessing how the public responds in online information-seeking practices during a pandemic situation. Studies conducted by Strzelecki [11], Effenberger et al [10], Springer et al [35], Husain et al [32], and Hu et al [36] are examples of this kind of study. Those studies used search engine query data to understand patterns of information-seeking behaviors, particularly in interpreting public interest toward the ongoing pandemic. Some studies [31,33,37] have also been designed specifically to understand essential health information searched for by the public as cases increased. In addition, these types of studies were also used to assess health risk communication strategies [30] and health risk perceptions [21].

For preliminary studies to assess the possibility of using search data for prediction purposes, most studies found high correlations between COVID-19 cases and online search data [4-6]. Some also exhibited highly correlated patterns in the preceding days [2] and weeks [3]. Therefore, internet searches have become a potential data source for predicting COVID-19–related metrics. However, limited studies are available that assess predictive performances of search volume models. Studies [12-14] conducted in the early months of the pandemic showed that proposed models that included search data performed better than those that did not include the search volumes. However, studies in the United States demonstrated low accuracy in model prediction [15] and variability in model performance among states and time periods [16].

Accordingly, in this study, we assessed the predictive performances of models that incorporated online search volumes. Data were aggregated into four subsets: 3, 6, 12, and 18 months of time series data. We intended to analyze whether search engine query data are important variables for inclusion in models for short- and long-term prediction of new daily COVID-19 cases and deaths. Results demonstrated promising use of NAVER search volumes for prediction tasks with higher feature effects in the first 6 months of the outbreak. Thus, this study provides an overview of using search data for predictive purposes in the context of a pandemic situation.

### Limitations

Analyses reported in this study only drew from perspectives of a demand-based infodemiological study. This means that this research examined the information-seeking behavior through search engine queries [9], which potentially reflect sudden changes in users’ online behaviors toward the ongoing pandemic [38]. Future analyses may need to take into account the supply-side analysis, incorporate other search engines’ data sets, as well as retrieve wider terms in order to capture wider infodemiological patterns in the population. In addition, other dynamic explanatory variables, such as health policy indices, may need to be included in the models to increase the model performance.

### Conclusions

NAVER search volumes were important variables with higher feature effects for predicting new daily COVID-19 cases, particularly in the first 6 months of the outbreak in South Korea. For longer periods, NAVER search volumes were still found to be important variables, although search term use should be considered, as more specific terms need to be used. Similar results were also found for death predictions. Likewise, GLMs with different types of distribution functions may be beneficial for use in the early stages of an outbreak. In longer periods, LR models with regularization may outperform GLMs as the number of possible explanatory variables that can be used in the models increases.

## Appendix

Multimedia Appendix 1 List of monthly top terms in the life and health category from NAVER; terms have been translated into English.

Multimedia Appendix 2 Correlations of new daily COVID-19 cases and deaths with explanatory variables in the training sets.

Multimedia Appendix 3 Important variables included in the models for predicting new daily COVID-19 cases.

Multimedia Appendix 4 Important variables included in the models for predicting new daily COVID-19 deaths.

## Abbreviations

AIC: Akaike information criterion
GLM: generalized linear model
LR: linear regression
MERS: Middle East respiratory syndrome
RMSE: root mean square error
