# Supplementary material for: Predicting New Daily COVID-19 Cases and Deaths Using Search Engine Query Data in South Korea From 2020 to 2021: Infodemiology Study
Source: J Med Internet Res. 2021 Dec 22;23(12):e34178. doi: 10.2196/34178 (PMC8698803; doi:10.2196/34178)
Supplement: Multimedia Appendix 1 [file jmir_v23i12e34178_app1.docx]

**Multimedia Appendix 1. Supplementary material**

List of monthly top terms in the life/health category from NAVER, terms have been translated into English

| Num | Term | Num | Term | | Num | Term | |
| --- | --- | --- | --- | --- | --- | --- | --- |
| April 2020 | | May 2020 | | June 2020 | | |  |
| 1. | Mask | 1. | Dental mask | | 1. | Splash mask | |
| 2. | Covid-19 | 2. | Mask | | 2. | Dental mask | |
| 3. | Kf94 mask | 3. | Yuhan Kimberly dental mask | | 3. | Mask | |
| 4. | Disposable mask | 4. | Domestic dental mask | | 4. | Kf94 mask | |
| 5. | Fine-dusk mask kf94 | 5. | Covid-19 | | 5. | Yuhan Kimberly dental mask | |
| 6. | Dental mask | 6. | Disposable mask | | 6. | 50 disposable masks | |
| 7. | Scales | 7. | 50 disposable masks | | 7. | Splash mask | |
| 8. | 50 disposable masks | 8. | Kf94 mask | | 8. | Scales | |
| 9. | Kf94 | 9. | Scales | | 9. | Domestic dental mask | |
| 10. | Thermometer | 10. | Children’s dental mask | | 10. | Wellkips splash mask | |
| July 2020 | | August 2020 | | September 2020 | | |  |
| 1. | Splash mask | 1. | Kf94 mask | | 1. | Mask strap | |
| 2. | Kf94 mask | 2. | Mask strap | | 2. | Kf94 mask | |
| 3. | Mask | 3. | Mask | | 3. | Foot bath | |
| 4. | Scales | 4. | Mask necklace | | 4. | Scales | |
| 5. | Kf-ad mask | 5. | Foot bath | | 5. | Mask | |
| 6. | Kf80 mask | 6. | Scales | | 6. | Mask necklace | |
| 7. | Dental mask | 7. | Kf94 | | 7. | Hand sanitizer | |
| 8. | Toothbrush | 8. | Kf80 mask | | 8. | Puppy feed | |
| 9. | Puppy feed | 9. | Hand sanitizer | | 9. | Starbucks tumbler | |
| 10. | Fine-dusk mask kf94 | 10. | Splash mask | | 10. | Massage gun | |
| October 2020 | | November 2020 | | December 2020 | | |  |
| 1. | Scales | 1. | Scales | | 1. | Christmas tree | |
| 2. | Foot bath | 2. | Foot bath | | 2. | Kf94 mask | |
| 3. | Kf94 mask | 3. | Kf94 mask | | 3. | Foot bath | |
| 4. | Leg tissue | 4. | Christmas tree | | 4. | Foot massager | |
| 5. | Mask strap | 5. | Foot massager | | 5. | Diary | |
| 6. | Mask | 6. | Mask | | 6. | Mask | |
| 7. | Dish drying rack | 7. | Leg tissue | | 7. | Leg tissue | |
| 8. | Puppy feed | 8. | Bidet | | 8. | Bidet | |
| 9. | Halloween costume | 9. | Scroll | | 9. | 2021 diary | |
| 10. | Starbucks tumbler | 10. | Starbucks tumbler | | 10. | Dishwasher detergent | |
| January 2021 | | February 2021 | | March 2021 | | |  |
| 1. | Mask | 1. | Mask | | 1. | Mask | |
| 2. | Foot bath | 2. | Scales | | 2. | Scales | |
| 3. | Kf94 mask | 3. | Foot bath | | 3. | Kf94 mask | |
| 4. | Diary | 4. | Kf94 mask | | 4. | Starbucks tumbler | |
| 5. | Leg tissue | 5. | Starbucks tumbler | | 5. | Bidet | |
| 6. | Starbucks playmobile | 6. | Happy call | | 6. | Waffle pan | |
| 7. | Delivery box | 7. | Kf94 mask, bird-beak type | | 7. | Car phone holder | |
| 8. | Starbucks tumbler | 8. | Holder | | 8. | Foot bath | |
| 9. | Snow chain | 9. | Waffle pan | | 9. | Mask strap | |
| 10. | Kf94 mask, bird-beak type | 10. | Leg tissue | | 10. | Kf94 mask, bird-beak type | |
| April 2021 | | May 2021 | | June 2021 | | |  |
| 1. | Mask | 1. | Scales | | 1. | Mask | |
| 2. | Scales | 2. | Mask | | 2. | Scales | |
| 3. | Kf94 mask | 3. | Kf94 mask | | 3. | Car phone holder | |
| 4. | Pop it | 4. | Car phone holder | | 4. | Mosquito net | |
| 5. | Dog food | 5. | Starbucks tumbler | | 5. | Dental mask | |
| 6. | Starbucks tumbler | 6. | Bidet | | 6. | Starbucks tumbler | |
| 7. | Dental mask | 7. | Kitchen towel | | 7. | Kf94 mask | |
| 8. | Bidet | 8. | Dog food | | 8. | Massage gun | |
| 9. | Mask strap | 9. | Dental mask | | 9. | Tumbler | |
| 10. | DC network | 10. | Pop it | | 10. | Car wireless charging cradle | |
| July 2021 | |  |  | |  |  | |
| 1. | Mask |  |  | |  |  | |
| 2. | Scales |  |  | |  |  | |
| 3. | Kf94 mask |  |  | |  |  | |
| 4. | Dental mask |  |  | |  |  | |
| 5. | Massage gun |  |  | |  |  | |
| 6. | Hayata whitening toothpaste | | | | | | |
| 7. | Starbucks tumbler |  |  | |  |  | |
| 8. | Pekoskin domestic dental droplet-blocking mask kf-ad | | | | | | |
| 9. | GP gangbliatiz kitchen detergent pump 500 ml lemon flavor | | | | | | |
| 10. | Tumbler |  |  | |  |  | |

Num, number.
