# Supplementary material for: Predicting New Daily COVID-19 Cases and Deaths Using Search Engine Query Data in South Korea From 2020 to 2021: Infodemiology Study
Source: J Med Internet Res. 2021 Dec 22;23(12):e34178. doi: 10.2196/34178 (PMC8698803; doi:10.2196/34178)
Supplement: Multimedia Appendix 2 [file jmir_v23i12e34178_app2.docx]

**Multimedia Appendix 2**

Correlations of new daily COVID-19 cases and deaths with explanatory variables in the training sets.

| Data subset^a^ | | | Explanatory variables^b^ | | | | | | | | | | | | | | | | | | | | | |
| --- | --- | --- | --- | --- | --- | --- | --- | --- | --- | --- | --- | --- | --- | --- | --- | --- | --- | --- | --- | --- | --- | --- | --- | --- |
|  | | | Case-based variables | | Google mobility | | | | | | Apple mobility | | NAVER search volumes | | | | | | | | | | | |
|  | | | 1 | 2 | 3 | 4 | 5 | 6 | 7 | 8 | 9 | 10 | 11 | 12 | 13 | 14 | 15 | 16 | 17 | 18 | 19 | 20 | 21 | 22 |
|  | | | | | | | | | | | | | | | | | | | | | | | | |
| **Correlation^c^ with new daily COVID-19 cases** | | | | | | | | | | | | | | | | | | | | | | | | |
|  | **Subset 1** | | | | | | | | | | | | | | | | | | | | | | | |
|  |  | *r* | 0.75 | 0.55 | –0.82 | –0.50 | 0.10 | –0.79 | –0.68 | 0.80 | –0.73 | –0.72 | 0.24 | 0.62 | 0.22 | 0.75 | 0.46 | 0.83 | 0.61 | 0.66 | 0.83 | 0.29 | 0.42 | 0.64 |
|  |  | *P* value | <.001 | <.001 | <.001 | <.001 | .38 | <.001 | <.001 | <.001 | <.001 | <.001 | .04 | <.001 | .07 | <.001 | <.001 | <.001 | <.001 | <.001 | <.001 | 0.01 | <.001 | <.001 |
|  | **Subset 2** | | | | | | | | | | | | | | | | | | | | | | | |
|  |  | *r* | 0.83 | 0.46 | –0.72 | –0.23 | 0.10 | –0.70 | –0.51 | 0.66 | –0.69 | –0.62 | 0.14 | 0.43 | 0.18 | 0.51 | 0.26 | 0.69 | 0.38 | 0.44 | 0.70 | 0.28 | 0.25 | 0.48 |
|  |  | *P* value | <.001 | <.001 | <.001 | .01 | .24 | <.001 | <.001 | <.001 | <.001 | <.001 | .08 | <.001 | .03 | <.001 | <.001 | <.001 | <.001 | <.001 | <.001 | <.001 | 0.002 | <.001 |
|  | **Subset 3** | | | | | | | | | | | | | | | | | | | | | | | |
|  |  | *r* | 0.85 | 0.33 | –0.53 | –0.06 | 0.06 | –0.38 | –0.39 | 0.34 | –0.45 | –0.36 | –0.05 | 0.11 | –0.09 | –0.07 | 0.30 | 0.19 | 0.25 | –0.13 | 0.39 | 0.04 | 0.47 | 0.18 |
|  |  | *P* value | <.001 | <.001 | <.001 | .31 | .28 | <.001 | <.001 | <.001 | <.001 | <.001 | .35 | .07 | .15 | .25 | <.001 | <.001 | <.001 | .02 | <.001 | .54 | <.001 | .002 |
|  | **Subset 4** | | | | | | | | | | | | | | | | | | | | | | | |
|  |  | *r* | 0.93 | 0.62 | –0.39 | –0.11 | –0.29 | –0.39 | –0.39 | 0.42 | –0.62 | –0.19 | –0.27 | –0.17 | –0.34 | –0.48 | 0.39 | –0.42 | 0.03 | –0.55 | 0.15 | –0.34 | 0.36 | –0.39 |
|  |  | *P* value | <.001 | <.001 | <.001 | .02 | <.001 | <.001 | <.001 | <.001 | <.001 | <.001 | <.001 | <.001 | <.001 | <.001 | <.001 | <.001 | 055 | <.001 | .001 | <.001 | <.001 | <.001 |
| **Correlation^c^ with new daily COVID-19 deaths** | | | | | | | | | | | | | | | | | | | | | | | | |
|  | **Subset 1** | | | | | | | | | | | | | | | | | | | | | | | |
|  |  | *r* | 0.65 | 0.62 | –0.66 | –0.23 | 0.35 | –0.66 | –0.62 | 0.65 | –0.72 | –0.73 | –0.23 | 0.32 | –0.20 | 0.39 | 0.72 | 0.55 | 0.15 | 0.20 | 0.56 | –0.14 | 0.55 | 0.22 |
|  |  | *P* value | <.001 | <.001 | <.001 | .05 | .002 | <.001 | <.001 | <.001 | <.001 | <.001 | .05 | .01 | .08 | <.001 | <.001 | <.001 | .19 | .08 | <.001 | .25 | <.001 | .06 |
|  | **Subset 2** | | | | | | | | | | | | | | | | | | | | | | | |
|  |  | *r* | 0.63 | 0.60 | –0.65 | –0.17 | 0.17 | –0.66 | –0.58 | 0.65 | –0.65 | –0.59 | 0.08 | 0.28 | 0.10 | 0.38 | 0.53 | 0.58 | 0.28 | 0.19 | 0.40 | 0.11 | 0.11 | 0.01 |
|  |  | *P* value | <.001 | <.001 | <.001 | .04 | .04 | <.001 | <.001 | <.001 | <.001 | <.001 | .31 | <.001 | .28 | <.001 | <.001 | <.001 | <.001 | .02 | <.001 | .17 | .20 | .92 |
|  | **Subset 3** | | | | | | | | | | | | | | | | | | | | | | | |
|  |  | *r* | 0.51 | 0.53 | –0.55 | –0.07 | 0.09 | –0.35 | –0.34 | 0.44 | –0.49 | –0.35 | 0.14 | 0.18 | 0.10 | 0.12 | 0.45 | 0.19 | 0.26 | 0.03 | 0.30 | 0.13 | 0.07 | –0.10 |
|  |  | *P* value | <.001 | <.001 | <.001 | .24 | .11 | <.001 | <.001 | <.001 | <.001 | <.001 | .02 | .002 | .08 | .04 | <.001 | .001 | <.001 | .65 | <.001 | .03 | .25 | .07 |
|  | **Subset 4** | | | | | | | | | | | | | | | | | | | | | | | |
|  |  | *r* | 0.71 | 0.72 | –0.45 | 0.05 | –0.25 | –0.45 | –0.43 | 0.54 | –0.62 | –0.26 | –0.10 | –0.06 | –0.20 | –0.39 | 0.46 | –0.34 | 0.05 | –0.45 | 0.13 | –0.24 | 0.09 | –0.48 |
|  |  | *P* value | <.001 | <.001 | <.001 | .31 | <.001 | <.001 | <.001 | <.001 | <.001 | <.001 | .02 | .22 | <.001 | <.001 | <.001 | <.001 | .26 | <.001 | .01 | <.001 | .05 | <.001 |

^a^Subsets 1 to 4: 3, 6, 12, and 18 months after the first case was reported in South Korea, respectively.

^b^1: daily new cases in the last 3 days; 2: daily new deaths in the last 3 days; 3: retail and recreation; 4: grocery and pharmacy; 5: parks; 6: transit stations; 7: workplaces; 8: residential areas; 9: driving; 10: walking; 11: 코로 나 바이러스 (coronavirus); 12: 코로나 바이러스 테스트 (coronavirus test); 13: 메르 스 (Middle East respiratory syndrome); 14: 마스크 (face mask); 15: 사회적 거리두기 (social distancing); 16: 신천지 (Shincheonji); 17: kf94 마스크 (kf94 mask); 18: 일회용 마스크 (disposable mask); 19: 온도계 (thermometer); 20: 손 소독제 (hand sanitizer); 21: 마스크스트랩 (mask strap); 22: Kf80 마스크 (kf80 mask).

^c^Moderate correlations range from *r*=0.50 to *r*≤0.69 and strong correlations range from *r*=0.70 to *r*≤1.
