# Supplementary material for: Predicting New Daily COVID-19 Cases and Deaths Using Search Engine Query Data in South Korea From 2020 to 2021: Infodemiology Study
Source: J Med Internet Res. 2021 Dec 22;23(12):e34178. doi: 10.2196/34178 (PMC8698803; doi:10.2196/34178)
Supplement: Multimedia Appendix 3 [file jmir_v23i12e34178_app3.docx]

**Multimedia Appendix 3**

Important variables included in the models for predicting new daily COVID-19 cases.

| Data subset^a^ | | Parameter estimates for the explanatory variables^b^ | | | | | | | | | | | | | | | | | | | | | | | | |
| --- | --- | --- | --- | --- | --- | --- | --- | --- | --- | --- | --- | --- | --- | --- | --- | --- | --- | --- | --- | --- | --- | --- | --- | --- | --- | --- |
|  |  | 1 | 2 | 3 | 4 | 5 | 6 | 7 | 8 | 9 | 10 | 11 | | 12 | 13 | 14 | 15 | 16 | | | 17 | 18 | 19 | 20 | 21 | 22 |
|  | | Case-based variables | | Google mobility | | | | | | Apple mobility | | | NAVER search volumes | | | | | | | | | | | | | |
|  | | | | | | | | | | | | | | | | | | | | | | | | | | |
| **Subset 1** | | | | | | | | | | | | | | | | | | | | | | | | | | |
|  | GLM1^c^ | 0.39 | —^d^ | — | — | — | — | — | — | — | — | — | | — | — | 5.41 | — | 1.44 | | | — | — | 8.58 | — | –2839.30 | –7.53 |
|  | GLM2^e^ | — | — | –0.11 | –0.03 | 0.01 | 0.09 | 0.04 | –0.05 | — | — | 0.02 | | –0.00 | 0.03 | –0.01 | 0.01 | 0.02 | | | 0.01 | 0.02 | 0.03 | –0.07 | –14.17 | –0.01 |
|  | GLM3^f^ | — | — | — | — | — | — | — | 0.35 | — | –0.04 | — | | — | 0.02 | — | — | 0.08 | | | — | — | — | — | — | — |
|  | LR1^g^ | 0.40 | –0.41 | — | –2.72 | 0.38 | — | –3.19 | — | — | 1.01 | 0.45 | | –0.19 | –0.57 | 3.40 | 0.62 | 1.43 | | | — | — | 8.09 | –0.19 | –1977.82 | –4.77 |
|  | LR2^h^ | 0.34 | — | 2.10 | –2.04 | — | — | –6.03 | — | — | 1.01 | 0.36 | | — | –0.89 | 6.91 | 0.47 | 1.52 | | | — | — | 8.47 |  | –2060.20 | –8.55 |
|  | LR3^i^ | 0.32 | –1.07 | — | –3.18 | 0.43 | — | –4.24 | –3.31 | — | 1.09 | 0.52 | | –0.38 | –0.81 | 5.65 | 0.93 | 1.45 | | | 0.63 | — | 8.54 | –0.38 | –2050.01 | –7.58 |
| **Subset 2** | | | | | | | | | | | | | | | | | | | | | | | | | | |
|  | GLM1 | 0.52 | — | — | — | — | — | — | — | 0.94 | — | — | | — | — | — | — | 1.74 | | | — | — | 6.68 | –0.75 | — | — |
|  | GLM2 | 0.00 | — | — | –0.02 | 0.02 | –0.07 | 0.03 | — | –0.01 | — | 0.01 | | 0.00 | 0.03 | 0.01 | 0.01 | 0.03 | | | 0.01 | — | 0.03 | –0.06 | 0.36 | — |
|  | GLM3 | — | — | –0.06 | — | 0.01 | — | — | — | — | –0.04 | — | | — | — | 0.02 | — | 0.06 | | | — | — | — | — | 0.44 | — |
|  | LR1 | 0.46 | –3.09 | –0.61 | — | — | — | –0.91 | — | 0.81 | — | 0.10 | | –0.26 | — | 1.01 | — | 1.63 | | | — | — | 6.59 | –0.57 | — | –1.07 |
|  | LR2 | 0.45 | –4.02 | — | — | — | — | –0.98 | –1.78 | 0.60 | — | — | | –0.78 | –0.24 | 3.82 | — | 1.54 | | | –1.00 | 1.20 | 6.66 | — | — | –3.86 |
|  | LR3 | 0.46 | –3.09 | –0.61 | — | — | — | –0.91 | — | 0.81 | — | 0.10 | | –0.26 | — | 1.01 | — | 1.63 | | | — | — | 6.59 | –0.57 | — | –1.07 |
| **Subset 3** | | | | | | | | | | | | | | | | | | | | | | | | | | |
|  | GLM1 | 0.51 | –3.45 | — | — | — | — | –2.03 | –7.08 | — | — | — | | — | — | 3.88 | — | | 1.74 | | –2.07 | — | 4.21 | –0.76 | 1.32 | — |
|  | GLM2 | 0.00 | 0.02 | –0.01 | — | 0.00 | –0.01 | –0.02 | –0.10 | –0.02 | 0.01 | 0.01 | | 0.00 | 0.01 | 0.05 | 0.00 | | 0.02 | | –0.03 | 0.03 | 0.00 | –0.05 | 0.03 | –0.01 |
|  | GLM3 | 0.00 | — | –0.01 | — | 0.01 | — | — | — | –0.02 | — | –0.07 | | 0.03 | 0.02 | — | — | | 0.04 | |  | 0.06 | — | — | 0.02 | — |
|  | LR1 | 0.48 | –2.64 | — | — | — | — | –1.66 | –5.18 | — | — | — | | — | — | 1.77 | — | | 1.47 | | –0.71 | 1.12 | 4.21 | –0.81 | 1.23 | –0.37 |
|  | LR2 | 0.51 | –2.52 | — | — | — | — | –1.87 | –6.34 | — | — | — | | — | — | 3.12 | — | | 1.58 | | –1.83 | 0.55 | 4.07 | –0.61 | 1.28 | — |
|  | LR3 | 0.48 | –2.64 | — | — | — | — | –1.66 | –5.18 | — | — | — | | — | — | 1.77 |  | | 1.47 | | –0.71 | 1.12 | 4.21 | –0.81 | 1.23 | –0.37 |
| **Subset 4** | | | | | | | | | | | | | | | | | | | | | | | | | | |
|  | GLM1 | 0.90 | — | 2.22 | — | — | –4.68 | –3.78 | –15.37 | –3.65 | 3.13 | — | | — | — | — | — | | | 2.40 | –2.72 | — | 6.51 | — | — | — |
|  | GLM2 | 0.00 | –0.01 | — | 0.01 | 0.00 | –0.02 | –0.02 | –0.09 | –0.07 | 0.05 | –0.02 | | 0.01 | — | –0.01 | — | | | 0.02 | –0.00 | 0.06 | 0.02 | –0.05 | 0.01 | — |
|  | GLM3 | 0.00 | — | — | 0.01 | — | –0.03 | –0.01 | –0.10 | –0.05 | 0.03 | –0.09 | | 0.03 | 0.04 | — | — | | | 0.03 |  | 0.08 | — | –0.02 | 0.02 | — |
|  | LR1 | 0.91 | –1.36 | 1.52 | 0.12 | –0.29 | –1.84 | –3.27 | –9.52 | –2.77 | 2.09 | — | | — | — | –0.83 | 0.53 | | | 2.02 | –2.46 | 1.68 | 5.06 | — | –0.27 | — |
|  | LR2 | 0.91 | –1.52 | 1.94 | — | –0.15 | –3.70 | –3.77 | –13.56 | –3.45 | 2.65 | 0.18 | | — | — | –2.07 | 0.45 | | | 1.89 | –2.57 | 2.57 | 5.92 | — | 0.42 | — |
|  | LR3 | 0.90 | –1.59 | 1.73 | 0.35 | –0.27 | –3.29 | –3.74 | –12.68 | –3.49 | 2.58 | 0.39 | | –0.33 | — | –1.95 | 0.52 | | | 2.01 | –2.66 | 2.31 | 5.76 | — | –0.48 | 0.39 |

^a^Subsets 1 to 4: 3, 6, 12, and 18 months after the first case was reported in South Korea, respectively.

^b^1: daily new cases in the last 3 days; 2: daily new deaths in the last 3 days; 3: retail and recreation; 4: grocery and pharmacy; 5: parks; 6: transit stations; 7: workplaces; 8: residential areas; 9: driving; 10: walking; 11: 코로 나 바이러스 (coronavirus); 12: 코로나 바이러스 테스트 (coronavirus test); 13: 메르 스 (Middle East respiratory syndrome); 14: 마스크 (face mask); 15: 사회적 거리두기 (social distancing); 16: 신천지 (Shincheonji); 17: kf94 마스크 (kf94 mask); 18: 일회용 마스크 (disposable mask); 19: 온도계 (thermometer); 20: 손 소독제 (hand sanitizer); 21: 마스크스트랩 (mask strap); 22: Kf80 마스크 (kf80 mask).

^c^GLM1: generalized linear model (GLM) with a normal distribution.

^d^This variable was not included in the model.

^e^GLM2: GLM with a Poisson distribution.

^f^GLM3: GLM with a negative binomial distribution.

^g^LR1: linear regression (LR) model with lasso regularization.

^h^LR2: LR model with adaptive lasso regularization.

^i^LR3: LR model with elastic net regularization.
