# Supplementary material for: Predicting New Daily COVID-19 Cases and Deaths Using Search Engine Query Data in South Korea From 2020 to 2021: Infodemiology Study
Source: J Med Internet Res. 2021 Dec 22;23(12):e34178. doi: 10.2196/34178 (PMC8698803; doi:10.2196/34178)
Supplement: Multimedia Appendix 4 [file jmir_v23i12e34178_app4.docx]

**Multimedia Appendix 4**

Important variables included in the models for predicting new daily COVID-19 deaths.

| Data subset^a^ | | Parameter estimates for the explanatory variables^b^ | | | | | | | | | | | | | | | | | | | | | | | | | |
| --- | --- | --- | --- | --- | --- | --- | --- | --- | --- | --- | --- | --- | --- | --- | --- | --- | --- | --- | --- | --- | --- | --- | --- | --- | --- | --- | --- |
|  |  | 1 | | 2 | 3 | 4 | 5 | 6 | 7 | 8 | 9 | 10 | 11 | 12 | 13 | 14 | 15 | 16 | 17 | | 18 | 19 | 20 | 21 | | 22 | |
|  | | Case-based variables | | | Google mobility | | | | | | Apple mobility | | NAVER search volumes | | | | | | | | | | | | | | |
|  | | | | | | | | | | | | | | | | | | | | | | | | | | | |
| **Subset 1** | | | | | | | | | | | | | | | | | | | | | | | | | | | |
|  | GLM1^c^ | —^d^ | | — | — | — | 0.06 | –0.17 | — | — | — | — | — | — | — | — | — | — | — | | — | — | — | — | | — | |
|  | GLM2^e^ | — | | — | — | — | — | — | — | — | — | –0.07 | — | — | 0.06 | — | — | — | –0.04 | | 0.11 | — | –0.17 | — | | — | |
|  | GLM3^f^ | — | | — | — | — | — | — | — | — | — | –0.07 | — | — | — | — | — | — | — | | — | — | — | — | | — | |
|  | LR1^g^ | — | | — | –0.05 | –0.06 | 0.03 | — | — | 0.07 | — | –0.03 | –0.00 | — | — | — | — | — | — | | — | — | — | 22.53 | | –0.02 | |
|  | LR2^h^ | — | | — | –0.10 | — | 0.03 | — | — | — | — | –0.03 | — | — | — | — | — | — | –0.00 | | — | — | — | — | | –0.02 | |
|  | LR3^i^ | — | | — | –0.05 | –0.06 | 0.03 | — | — | 0.07 | — | –0.03 | –0.00 | — | — | — | — | — | — | | — | — | — | 22.53 | | –0.02 | |
| **Subset 2** | | | | | | | | | | | | | | | | | | | | | | | | | | | |
|  | GLM1 | — | | 0.35 | — | — | — | –0.11 | — | — | — | — | — | — | — | — | — | — | — | | — | — | — | — | | — | |
|  | GLM2 | — | | — | — | — | — | — | — | — | –0.05 | –0.03 | 0.02 | — | 0.07 | — | — | — | — | | — | — | — | –0.08 | | — | |
|  | GLM3 | — | | 0.10 | — | — | — | –0.03 | — | — | — | –0.05 | — | — | — | — | — | — | — | | — | — | — | –0.52 | | — | |
|  | LR1 | 0.00 | | 0.14 | –0.08 | –0.01 | 0.01 | — | — | 0.01 | –0.01 | — | — | 0.04 | –0.00 | 0.06 | — | — | — | | –0.06 | 0.03 | –0.00 | –0.26 | | –0.07 | |
|  | LR2 | — | | 0.11 | –0.09 | — | 0.01 | — | — | — | –0.01 | — | — | 0.03 | — | 0.12 | — | — | –0.02 | | –0.06 | — | — | — | | –0.10 | |
|  | LR3 | 0.00 | | 0.14 | –0.08 | –0.01 | 0.01 | — | — | 0.01 | –0.01 | — | — | 0.04 | –0.00 | 0.06 | — | — | — | | –0.06 | 0.03 | –0.00 | –0.26 | | –0.07 | |
| **Subset 3** | | | | | | | | | | | | | | | | | | | | | | | | | | | |
|  | GLM1 | — | | 0.36 | –0.05 | — | — | — | — | — | –0.02 | — | — | — | — | — | — | — | — | — | | — | — | — | | — | |
|  | GLM2 | — | | 0.06 | –0.08 | 0.03 | 0.01 | — | 0.04 | 0.08 | –0.03 | 0.02 | — | 0.02 | — | — | — | — | — | — | | — | –0.05 | — | | — | |
|  | GLM3 | — | | 0.08 | –0.09 | 0.02 | 0.01 | 0.04 | 0.04 | 0.13 | –0.01 | — | — | — | — | — | — | — | — | — | | — | — | — | | — | |
|  | LR1 | 0.00 | | 0.18 | –0.07 | 0.02 | 0.01 | — | 0.04 | 0.07 | –0.01 | 0.01 | — | 0.04 | — | 0.07 | 0.00 | — | — | –0.08 | | 0.01 | –0.01 | — | | –0.05 | |
|  | LR2 | 0.00 | | 0.16 | –0.08 | 0.02 | 0.01 | — | 0.04 | 0.07 | –0.01 | 0.01 | — | 0.05 | — | 0.09 | — | — | — | –0.10 | | 0.02 | –0.02 | — | | –0.05 | |
|  | LR3 | 0.00 | | 0.18 | –0.07 | 0.02 | 0.01 | — | 0.04 | 0.07 | –0.01 | 0.01 | — | 0.04 | — | 0.07 | 0.00 | — | — | –0.08 | | 0.01 | –0.01 | — | | –0.05 | |
| **Subset 4** | | | | | | | | | | | | | | | | | | | | | | | | | | | |
|  | GLM1 | 0.01 | 0.47 | | — | — | — | — | 0.04 | 0.34 | — | — | — | — | — | — | — | — | — | | — | — | — | | — | | –0.05 |
|  | GLM2 | 0.00 | 0.02 | | — | — | –0.00 | — | — | 0.04 | –0.03 | — | –0.04 | 0.02 | — | 0.04 | — | — | — | | 0.03 | — | — | | — | | –0.07 |
|  | GLM3 | 0.00 | 0.04 | | — | — | — | — | — | 0.05 | –0.04 | 0.02 | –0.04 | 0.03 | — | 0.05 | — | — | — | | — | — | — | | 0.01 | | –0.06 |
|  | LR1 | 0.01 | 0.44 | | — | –0.02 | –0.00 | 0.05 | 0.02 | 0.40 | — | –0.00 | — | 0.04 | — | — | — | 0.01 | –0.04 | | –0.05 | 0.01 | 0.02 | | –0.01 | | –0.01 |
|  | LR2 | 0.01 | 0.44 | | — | –0.02 | — | 0.06 | 0.03 | 0.45 | — | — | — | 0.05 | — | — | — | — | –0.04 | | –0.05 | — | 0.02 | | –0.01 | | — |
|  | LR3 | 0.01 | 0.44 | | — | –0.02 | –0.00 | 0.05 | 0.02 | 0.40 | — | –0.00 | — | 0.04 | — | — | — | 0.01 | –0.04 | | –0.05 | 0.01 | 0.02 | | –0.01 | | –0.01 |

^a^Subsets 1 to 4: 3, 6, 12, and 18 months after the first case was reported in South Korea, respectively.

^b^1: daily new cases in the last 3 days; 2: daily new deaths in the last 3 days; 3: retail and recreation; 4: grocery and pharmacy; 5: parks; 6: transit stations; 7: workplaces; 8: residential areas; 9: driving; 10: walking; 11: 코로 나 바이러스 (coronavirus); 12: 코로나 바이러스 테스트 (coronavirus test); 13: 메르 스 (Middle East respiratory syndrome); 14: 마스크 (face mask); 15: 사회적 거리두기 (social distancing); 16: 신천지 (Shincheonji); 17: kf94 마스크 (kf94 mask); 18: 일회용 마스크 (disposable mask); 19: 온도계 (thermometer); 20: 손 소독제 (hand sanitizer); 21: 마스크스트랩 (mask strap); 22: Kf80 마스크 (kf80 mask).

^c^GLM1: generalized linear model (GLM) with a normal distribution.

^d^This variable was not included in the model.

^e^GLM2: GLM with a Poisson distribution.

^f^GLM3: GLM with a negative binomial distribution.

^g^LR1: linear regression (LR) model with lasso regularization.

^h^LR2: LR model with adaptive lasso regularization.

^i^LR3: LR model with elastic net regularization.
